# Does overweight and obesity have an impact on delivery mode and peripartum outcome in breech presentation? A FRABAT cohort study

**DOI:** 10.1007/s00404-024-07403-7

**Published:** 2024-03-18

**Authors:** Lukas Jennewein, Lena Agel, Samira Catharina Hoock, Anna Elisabeth Hentrich, Frank Louwen, Nadja Zander

**Affiliations:** Department of Obstetrics and Perinatal Medicine, University Hospital, Goethe University Frankfurt, 60590 Frankfurt, Germany

**Keywords:** Obesity, Maternal BMI, Breech delivery, Perinatal outcome

## Abstract

**Purpose:**

Obesity is a worldwide and growing issue affecting women in childbearing age, complicating surgical procedures as well as pregnancy. Through a reduction of not necessarily required cesarean deliveries—for instance in pregnancies with breech presentation—obesity mediated and surgery-associated morbidity might be contained. Date on the impact of maternal BMI in vaginally attempted breech delivery is not existing. To give insight into whether an elevated BMI leads to an increased perinatal morbidity in vaginally intended deliveries out of breech presentation, we analyzed delivery outcome of laboring women with a singleton baby in breech presentation with overweight and obesity (BMI ≥ 25 kg/m^2^) in comparison to women with a BMI of below 25 kg/m^2^.

**Methods:**

Based on data from January 2004 to December 2020, a cohort study was performed on 1641 women presenting with breech presentation at term (> 37 weeks). The influence of maternal BMI on perinatal outcome was analyzed with Chi^2^ testing for group differences and logistic regression analysis. Patients with a hyperglycemic metabolism were excluded from the study.

**Results:**

Fetal morbidity was not different when patients with a BMI of ≥ 25 kg/m^2^ (PREMODA morbidity score 2.16%) were compared to patients with a BMI of below 25 kg/m^2^ (1.97%, *p* = 0.821). Cesarean delivery rates were significantly higher in overweight and obese women with 43.9% compared to 29.3% (*p* < 0.0001). BMI and cesarean delivery were significantly associated in a logistic regression analysis (Chi^2^ coefficient 18.05, *p* < 0.0001). In successful vaginal deliveries out of breech presentation, maternal perineal injury rates (vaginal birth in normal-BMI women 48.4%; vaginal birth in overweight and obese women: 44.2%; *p* = 0.273) and rates of manually assisted delivery (vaginal birth in normal-BMI women: 44.4%; vaginal birth in obese and overweight women: 44.2%; *p* = 0.958) were not different between BMI groups.

**Conclusions:**

Obesity and overweight are not associated with peripartum maternal or newborn morbidity in vaginally attempted breech delivery, if the patient cohort is thoroughly selected and vaginal breech delivery is in an upright maternal position. Reduction of cesarean delivery rates, especially in overweight and obese women might, have an important positive impact on maternal and newborn morbidity.

## What does this study add to the clinical work

**Table:** 

Elevated maternal BMI or obesity is not a contraindication for a vaginal delivery attempt in breech presentation. Through reducing cesarean section rates especially in obese women, maternal morbidity can be decreased.	

## Introduction

Maternal obesity is a proven risk factor for maternal and neonatal morbidity [1, 2] and obesity numbers and associated complications are increasing worldwide [3]. This applies in particular to women of childbearing age. According to the WHO, in 2016, more than 39% of people over the age of 18 were overweight and 13% were even obese. In the US, more than 60% of reproductive age population are now overweight or obese [4]. Obese patients are known to be significantly more at risk for surgical complications compared to non-obese patients [5]. In terms of birth and labor, obese women profit from a restrictive management concerning cesarean sections. Even for the offspring, negative effects of a cesarean section have been extensively reported [6].

Pregnant women with a breech presentation at term often receive a cesarean section as mode of delivery. Since only 4% of all fetuses are in breech position [7], the expertise in vaginal breech delivery is scarce. Triggered by the term breech trial (TBT) by Hannah et al. in 2000 in many “high-income countries”, the planned cesarean section is still recommended to pregnant women as the safest method of delivery [8–11]. Today, the vaginal birth attempt is a safe, established, and guideline-supported mode of delivery for breech presentation [12–14]. Vaginal birth in upright maternal position has an excellent outcome for mother and child [15]. Also, a suspected high birth weight, which is more likely in obese women, is not an exclusion criterion for vaginal birth attempt [16]. It seems obvious, that reducing cesarean section rates comes with a decrease in maternal morbidity, such as postpartum bleeding, uterine rupture, and placentation disorders in subsequent pregnancies [17]. Obese women might benefit even more from avoiding unnecessary cesarean section.

There are only little data concerning vaginal delivery in cases with breech presentation an obesity. For preterm labor, obesity is described as a risk factor for adverse fetal outcome [18].

There are also criteria that are described as disadvantageous for the attempt to deliver vaginally, but which are not evidence-based. This includes, for example, an increased maternal body mass index (BMI).

However, obese women with a breech presentation at term cannot be given scientifically based recommendations which birth mode to prefer. So far, there is no study existing that considers the maternal, neonatal, and peripartum outcome in obese pregnant women with breech presentation at term.

To improve the counseling of these pregnant women and thus to promote patient safety consistent evidence is needed regarding the delivery mode at term (37 weeks to 42 weeks of gestation).

For this purpose, we conducted a cohort study from 2004 to 2020 investigating the impact of obesity on the peripartum outcome of mother and infant in attempted spontaneous delivery with breech presentation.

## Materials and methods

### Patient cohort and patient selection

The FRABAT Study is a prospective register study, including patients since January 2004 with breech at term (> 37 weeks) at the Goethe University Hospital Frankfurt. This study is a retrospectively performed analysis of these data, including the time period of 2004 until December 2020. Since the data to be evaluated were collected from the normal patient files and all patients were treated according to the standards of the obstetrics department, no written patient consent was required. The procedure and the protocol were approved by the ethics committee of the Goethe University Hospital Frankfurt, Germany (470/11). All data were anonymized. Data regarding the patient histories, the treatment of the patients and the outcome parameters of the neonates were obtained using the hospitals documentation system as well as the state database “Perinatalerhebung Hessen”. Inclusion criteria were patients who presented at 34–36 weeks of gestation with breech presentation at our outpatient clinic for delivery planning and registration. The standardized counseling process as well as the clinical management has been published in several studies of the FRABAT study collective [15, 16, 19–23].

Out of a total of 2472 patients in this period presenting to our hospital for delivery with breech presentation, we could include 1641 patients in this study analysis.

These patients intended to give birth spontaneously and did not meet the exclusion criteria such as planned cesarean delivery, uterine abnormalities, placental abnormalities, severe maternal morbidity (pre-eclampsia, HELLP-syndrome, and severe internal diseases), pelvic deformity, fetal malformation, estimated fetal weight less than 2.5 kg, or disproportional growth of the fetus. Patients with an unstable blood sugar profile while having gestational diabetes or patients with a diabetes type II were excluded from the study.

**Fig. 1:**
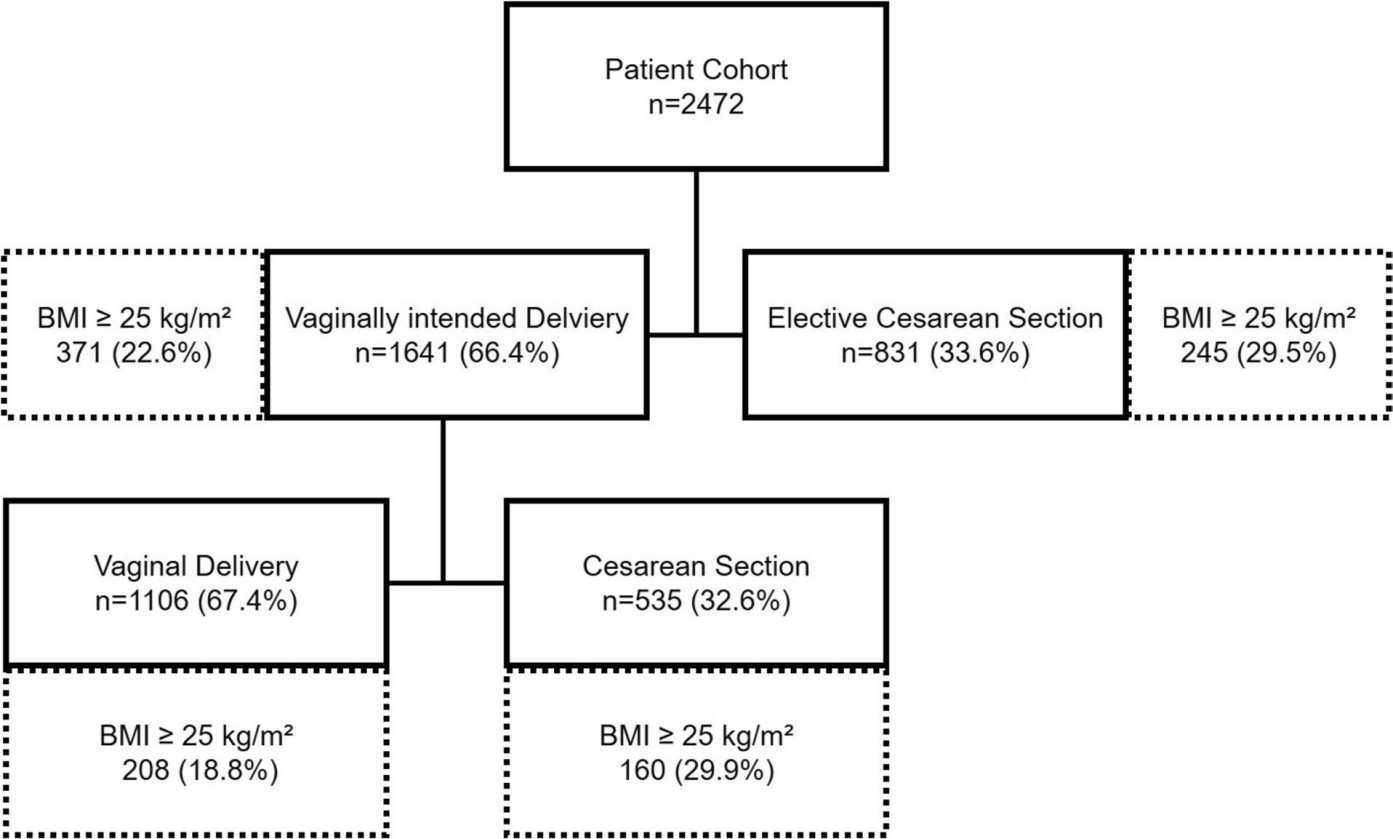
Flowchart displaying the study cohort with delivery outcome

Out of all included patients, two BMI groups were generated. One group comprised all patients with a BMI ≥ 25 kg/m^2^, the other group consisted of all patients with a BMI < 25 kg/m^2^. The allocation of the study population to the groups was based on BMI before pregnancy. Figure 1 shows the study design and the number of the patients assigned to each group.

### Statistical analyses

Grouped variables were tested for normal distribution using Kolmogorov–Smirnov test. Where values were normally distributed, epidemiological data were tested using the Student’s *t* test. Non-parametric data were tested using the Pearson’s χ^2^ test for group differences. A logistic regression analysis with reverse prediction was performed based on χ^2^ testing. A p value of below 0.05 was regarded as statistically significant.

All statistical analysis has been performed using JMP software 17.0 (SAS Institute, Cary, USA).

### Primary outcome measures

Newborn morbidity was the primary outcome. It was measured using a modified PREMODA score. One of the following five items (or more) had to apply to fulfill the criteria for newborn morbidity: (1) 5 min APGAR score; (2) fetal birth injury, e.g., humerus fracture or paresis of the plexus brachialis; (3) intubation period of more than 24 h; (4) neurological deficit; (5) stay on the neonatal intensive care unit more than 4 days. This score was applied from the PREMODA study. [24] After inspecting each PROMODA case, those not possibly related to the birth mode were excluded (e.g., infection, hypoglycemia, and transient problems with adaption), resulting in the score “PREMODA potentially related to birth mode”.

### Secondary outcome measures

We analyzed rate of cesarean delivery and epidural anesthesia. In vaginally succeeded deliveries, we examined rate of manual assistance and maternal outcome in terms of perineal injuries including high-grade perineal tears (grades III, IV).

## Results

2472 patients were counseled on their delivery. 831 women (33.6%) either met exclusion criteria for vaginal attempted delivery or opted for cesarean section (by choice). The portion of women receiving an elective cesarean section with a BMI ≥ 25 kg/m^2^ was 29.5% and significantly larger compared to women intending vaginal delivery (22.6%, *p* > 0.0001) (see Fig. 1).

1641 participants opted for the vaginal birth attempt. Mean age was 32.2 years (± 4.4 years standard deviation), and mean BMI was 23.1 kg/m^2^ (± 3.9 kg/m^2^ standard deviation). Rate of cesarean deliveries was 32.6%. For more descriptive data, see Table 1. Two groups were generated by separating at BMI 25 kg/m^2^, resulting in a normal-BMI group (nBMI) with 1270 cases and a high-BMI group (hBMI) with 371 cases.

**Table 1:** Descriptive data, whole cohort of vaginally intended deliveries

Variable	*N* = 1641
Age (years ± SD)	32.3 ± 4.4
Weight (kg ± SD)	65.8 ± 11.8
Height (cm ± SD)	168.7 ± 6.3
BMI (kg/m^2^ ± SD)	23.1 ± 3.9
BMI ≥ 25 kg/m^2^ (kg/m^2^ ± SD)	371 (22.6%)
Pregnancy duration (weeks ± SD)	39.5 ± 1.2
Primiparity (*N*, %)	952 (58.0%)
Epidural (*N*, %)	881 (53.7%)
Cesarean section (*N*, %)	535 (32.6%)
Fetal birth weight (grams ± SD)	3359 ± 420

### Primary outcome

Newborn outcome (modified PREMODA score) was not significantly different between BMI groups with 1.97% in nBMI and 2.16% in hBMI, *p* = 0.821. Single parameters calculated within the modified PREMODA score were not significantly different either: 5 min. APGAR score < 4 rate was 0.55% in nBMI and 0.81% in hBMI, *p* = 0.575. 4.4% of newborns had to stay in the neonatal intensive care unit (NICU) for more than 4 days in nBMI compared to 6.7% in hBMI, *p* = 0.076. Fetal birth injuries were equally distributed between groups: nBMI: 0.79%, hBMI 0.54%, *p* = 0.621. Rates of newborn intubation were not significantly different (nBMI: 0.87%, hBMI: 0.81, *p* = 0.621). There was no significant difference in neurological deficits (nBMI: 0.39%, hBMI: 0.81%), *p* = 0.313) (see Table 2).

**Table 2:** Vaginally intended deliveries, comparison between normal-BMI group (nBMI) and high-BMI group (hBMI)

Variable	NBMI < 25 kg/m^2^ *N* = 1270	HBMI ≥ 25 kg/m^2^ *N* = 371	*p* value
Age (years ± SD)	32.3 ± 4.3	32.4 ± 4.7	0.559
Pregnancy duration (weeks ± SD)	39.5 ± 1.2	39.6 ± 1.2	0.034
Primiparity (*N*, %)	742 (58.4%)	210 (56.6%)	0.532
Frank breech presentation (*N*, %)	816 (64.3%)	221 (59.6%)	0.099
Insulin-treated gestational diabetes (*N*, %)	16 (1.3%)	18 (4.9%)	< 0.0001
Diet-treated gestational diabetes (*N*, %)	44 (3.5%)	21 (5.7%)	0.056
Cesarean delivery (*N*, %)	372 (29.3%)	163 (43.9%)	< 0.0001
Epidural anesthesia (*N*, %)	696 (54.8%)	185 (49.9%)	0.093
Fetal birth weight (grams ± SD)	3335.3 ± 407	3440.1 ± 454	< 0.0001
5 min APGAR < 4 (*N*, %)	7 (0.55%)	3 (0.81%)	0.575
Nicu > 4 days (*N*, %)	56 (4.4%)	25 (6.7%)	0.076
Fetal birth injury (*N*, %)	10 (0.79%)	2 (0.54%)	0.621
Intubation > 24 h (*N*, %)	11 (0.87%)	6 (1.6%)	0.241
Neurological deficit (*N*, %)	5 (0.39%)	3 (0.81%)	0.313
Newborn infection (*N*, %)	46 (3.6%)	19 (5.1%)	0.193
PREMODA (*N*, %)	62 (4.9%)	28 (7.6%)	0.052
PREMODA potentially related to birth mode (*N*, %)	25 (1.97%)	8 (2.16%)	0.821

### Secondary outcome

**Fig. 2:**
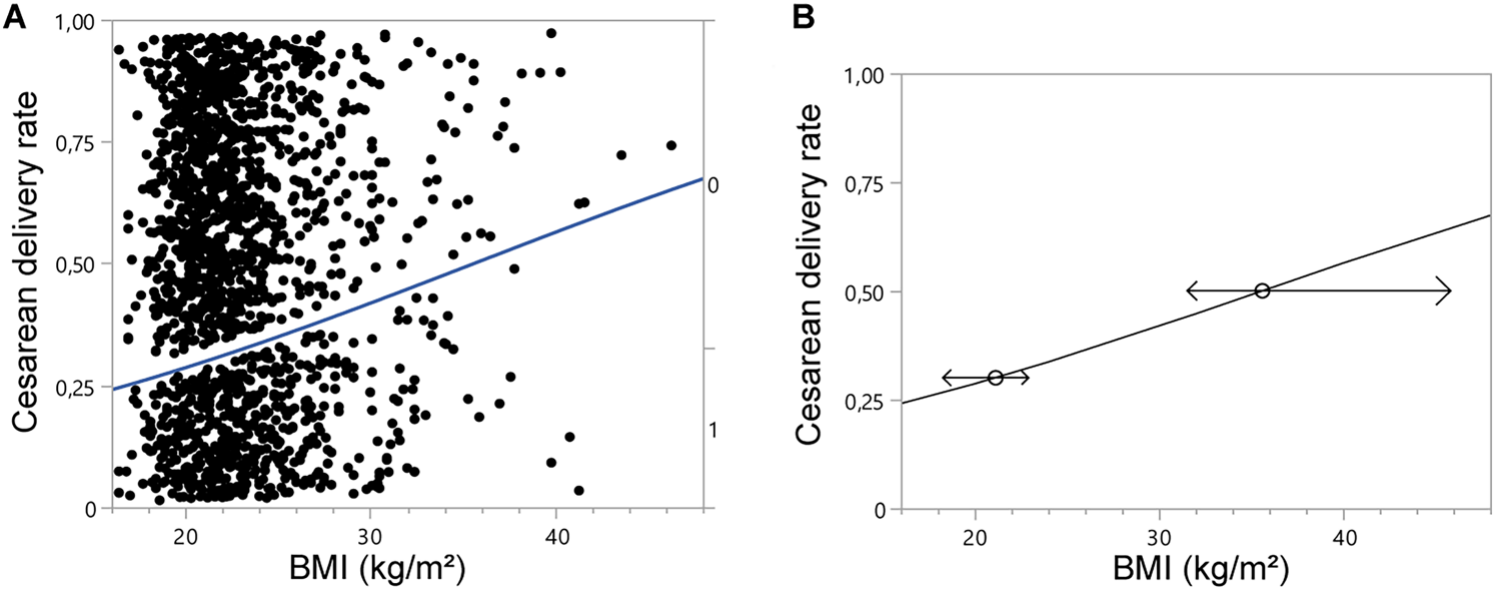
Logistic regression analysis BMI vs. cesarean section rate. Logistic regression analysis was performed plotting the cesarean section rates on the continuous variable BMI (kg/m^2^), displayed in A with each dot representing one patient. *R*^2^(U) = 0.0087 Chi^2^ 18.05, *p* < 0.0001, Odds ratio per unit: 1.058 B) Reverse prediction calculation of the BMI with a Cesarean delivery rate of 30%: 21.15 kg/m^2^ (95% CI 18.2–22.9 kg/m^2^) and 50%: 35.63 kg/m^2^ (95% CI 31.5–45.9 kg/m^2^)

Cesarean section rate was significantly higher in hBMI with 43.9% compared to 29.3% in nBMI with a p value of below 0.0001 (Table 1). In a logarithmic regression analysis, cesarean section rate was significantly associated with BMI (Chi^2^ 18.05, *R*^2^ = 0.0087, *p* > 0.0001). Odds ratio per unit was 1.058 (See Fig. 2). A reverse prediction in our logarithmic regression model revealed a BMI of up to 21.1 kg/m^2^ (95% CI 18.3–22.9 kg/m^2^) resulting in a cesarean section rate of 30%. A cesarean section rate of above 50% was associated with a BMI of above 35.6 kg/m^2^ (95% CI 31.5–45.9 kg/m^2^, Fig. 2). Rates of epidural anesthesia did not differ significantly between groups (nBMI: 54.8%, hBMI: 49.9%, *p* = 0.093). Birth weight was slightly but significantly higher in hBMI with a mean of 3440 g (± 454 g) compared to nBMI with a mean of 3335 g (± 407 g) with a p value of below 0.0001. Also, pregnancy duration showed a significant difference with a longer pregnancy in hBMI pregnancies (39.6 ± 1.2 weeks compared to 39.5 ± 1.2 in nBMI, *p* = 0.034). There was a significantly higher rate of insulin-treated gestational diabetes in women with a high BMI (hBMI: 3.9% compared to nBMI: 1.3%, *p* < 0.0001). See Table 2.

We applied the WHO classification on obesity and analyzed for cesarean section rates: In normal weighted women, CS rate was 29.1%. In overweight women (BMI 25–29.9 kg/m^2^) CS rate was 42.6%. In women with obesity I° (BMI 30–34.9 kg/m^2^), CS rate was 50%. In women giving birth with an obesity II° (BMI 35–39.9 kg/m^2^), CS rate was 30%. The CS rate in the group of women with a BMI of above 40 kg/m^2^ (obesity III°) was 28.6%. A *p* value of < 0.0001 was calculated for over all group differences in this analysis. See Table 3.

**Table 3:** Vaginally intended deliveries, cesarean section rates in BMI groups according to the WHO obesity classification

BMI group (who)	*N*	Cesarean section rate
Normal weight (< 25 kg/m^2^)	1271	29.1% (370)
Overweight (25–29.9 kg/m^2^)	265	42.6% (113)
Obesity I° (30–34.9 kg/m^2^)	78	50.0% (39)
Obesity II° (35–39.9 kg/m^2^)	20	30.0% (6)
Obesity III° (≥ 40 kg/m^2^)	7	28.6% (2)

In vaginally succeeded deliveries, rate of necessary manual assistance was equally distributed between BMI groups (vag nBMI 44.4%, vag hBMI 44.2%, *p* = 0.958). Mean birth duration was equally long with 385.6 ± 301 min in vag nBMI and 391 ± 367 min in vag hBMI (*p* = 0.441). Perineal injuries were not significantly different (48.5% in vag nBMI and 44.2% in vag hBMI, *p* = 0.273). Also, rates of high-grade perineal tears were equally distributed (vag nBMI: 2.01%, vag hBMI 1.92%, *p* = 0.938, Table 4).

**Table 4:** Subgroup of vaginally succeeded deliveries

Variable	VAG NBMI < 25 *N* = 898	VAG HBMI ≥ 25 *N* = 208	*p *Value
Manual assistance (*N*, %)	399 (44.4%)	92 (44.2%)	0.958
Assistance with arm delivery/Louwen maneuver (*N*, %)	224 (24.9%)	60 (28.9%)	0.246
Assistance with head delivery/rank nudge (*N*, %)	354 (39.4%)	79 (38.0%)	0.701
Epidural anesthesia (*N*, %)	456 (50.8%)	89 (42.8%)	0.045
Birth duration (minutes, mean ± SD)	385.6 ± 301	391.0 ± 367	0.441
Perineal injury (*N*, %)	435 (48.4%)	92 (44.2%)	0.273
High-grade perineal injury (degree III and IV)	18 (2.01%)	4 (1.92%)	0.938
PREMODA (*N*, %)	43 (4.79%)	12 (5.77%)	0.558
PREMODA potentially related to birth mode (*N*, %)	22 (2.45%)	5 (2.40%)	0.969

## Discussion

Obesity is a worldwide, growing problem which predominantly affects women in childbearing age [4]. High cesarean delivery numbers cause increased perioperative and long-term morbidity in women and newborns [25, 26]. Especially, women with obesity are prone to experience complications from surgical procedures. Thus, avoiding cesarean delivery when not particularly necessary might lower cesarean section associated morbidity rates in obese women. Also, newborns would benefit from a natural birth [27]. Fetal breech presentation poses an indication for cesarean section in the majority of obstetric departments, even though national guidelines emphasize the security of vaginal delivery in terms of maternal and newborn perinatal morbidity [12, 14]. If a high body mass index leads to an increased perinatal morbidity in vaginally intended breech delivery never has been explicitly examined before. This study was designed to shed light into the influence of the preconceptional BMI on perinatal outcome in vaginal attempted breech delivery. In our cohort study, we compare deliveries of women with breech presentation with a BMI of below 25 kg/m^2^ (normal-BMI group, nBMI) to delivering women who exceed the 25 kg/m^2^ BMI bench mark (high-BMI group, hBMI). Our data show that short-term perinatal morbidity is not impacted by the maternal body mass index. Rates in our modified PREMODA score are not significantly different between BMI groups (nBMI: 1.97%, hBMI: 2.16%, and *p* = 0.821, Table 1). Moreover, a logistic regression analysis examining the association of the body mass index as a continuous variable on the modified PREMODA score did not reveal an association (*R*^2^ = 0.0007, Odd ratio per unit: 1.021, *p* = 0.626, data not shown). Of note, patients with non-optimal blood sugar daily profile with gestational diabetes or diabetes mellitus type I or II were excluded from the study. Hence, we conclude that patients with a physiological blood sugar stability during pregnancy do not have an association between their BMI and newborn morbidity in vaginal attempted breech delivery.

As a secondary outcome measure, we analyzed the impact of maternal BMI on cesarean section (CS) rates in vaginally attempted breech deliveries. Here, we see a significant difference: Patients with a BMI below 25 kg/m^2^ had a CS rate of 29.3%. Obese patients (BMI ≥ 25 kg/m^2^) received an emergency cesarean delivery in 43.9% of cases (*p* < 0.0001, Table 1).

Our cutoff at 25 kg/m^2^ brings overweight (BMI 25 kg/m^2^–29.9 kg/m^2^) and obese (BMI ≥ 30 kg/m^2^) into one group which is compared to patients who are not overweight (BMI < 25 kg/m^2^). Risks mediated through obesity might thus be underrepresented in our analysis. To overcome this issue, we performed our statistics also with a BMI cutoff at 30 kg/m^2^. Here, we see equivalent results (data not shown), but the quality of statistical tests is limited, since group size differences are higher. To validate our findings, that BMI and CS rates are significantly associated, we performed a logistic regression analysis based on the continuous variable BMI. In this logistic regression model, a significant positive correlation of the maternal BMI and CS probability was detected with a 1.058-fold increase in CS probability per BMI unit (*p* < 0.0001). A reverse prediction calculation revealed a probability for CS of 50% when a BMI of 35.6 kg/m^2^ was apparent. When the study cohort was categorized using the WHO obesity classification, we see elevated cesarean section rates in women with overweight and obesity I°. Women with obesity II° and III° had CS rate of 30 and 28.6%, respectively (Table 3). This result is probably due to very low patient numbers in WHO grade II and III obesity groups in our analysis. The few women deciding on a vaginal birth attempt with a BMI of above 35 kg/m^2^ might have a strong personal need for a natural birth. Additionally, the portion of multiparous women was greater in the WHO grade III° group which impacts CS probability directly (data not shown), putting these results into perspective.

Again, fetal morbidity was not associated with an elevated BMI (data not shown). It is worth going in to labor, even if the odds for cesarean delivery are high. The positive effects of contractions and birth hormones on fetal adjustment after delivery are well documented [28, 29]. Pregnant women who wish to attempt vaginal birth should be given the chance to do so if evidence-based contraindications are excluded.

In our subgroup analysis of vaginally succeeded deliveries, we did not see a difference in rates of manually assisted delivery, maternal morbidity in terms of perineal lacerations, or newborn morbidity (Table 4). These findings suggest that obesity does not have an impact on complication rates such as shoulder dystocia in breech or maternal birth injuries in the second stage of labor.

A limitation of this study is that it is single-center based. Inclusion criteria and clinical management of cases with breech presentation are widely different within the obstetric clinical landscape. For instance, in our cohort, women give birth in an upright maternal position which decreases perinatal morbidity and rates of manual assistance [15]. This is due to not unified guidelines on breech presentation [30] and individually developed expertise of the managing obstetricians. Unified guidelines and evidence-based information are desperately needed to improve peripartum care for pregnant women with breech presentation.

Our data prove that overweight or obesity are not indicators on which a choice on the mode of delivery should be solely based on in pregnant women with breech presentation. The prerequisite is a selected cohort in which pathological diabetic metabolism is excluded, since it has convincingly been demonstrated that maternal and newborn morbidity is reduced by effective diabetes treatment [31]. In our cohort, patients with a gestational diabetes are only recommended for vaginal delivery if blood sugar values are within physiological range over the course of several weeks before the estimated due date.

The growth of evidence delivered by this study is able to reduce cesarean delivery rates in breech presentation. This might have a positive impact on perinatal morbidity. Furthermore, pregnancy counseling in cases with breech presentation might rely on our data to offer evidence-based information to women preferring vaginal delivery.

## Data Availability

Data will be available on request.
